# Amphibians on the hotspot: Molecular biology and conservation in the South American Atlantic Rainforest

**DOI:** 10.1371/journal.pone.0224320

**Published:** 2019-10-23

**Authors:** Cesar R. L. Amaral, Anna C. S. Chaves, Vitor N. T. Borges Júnior, Filipe Pereira, Bruna M. Silva, Dayse A. Silva, António Amorim, Elizeu F. Carvalho, Carlos F. D. Rocha

**Affiliations:** 1 Departamento de Ecologia, Instituto de Biologia Roberto Alcantara Gomes, Universidade do Estado do Rio de Janeiro, Rio de Janeiro, Brazil; 2 Interdisciplinary Centre of Marine and Environmental Research (CIIMAR), University of Porto, Porto, Portugal; 3 Instituto de Patologia Molecular e Imunologia (IPATIMUP) / Instituto de Investigação e Inovação em Saúde, Universidade do Porto, Porto, Portugal; 4 Faculdade de Ciências, Universidade do Porto, Porto, Portugal; Universidad de Sevilla, SPAIN

## Abstract

Amphibians are the focus of a recent debate and public attention owing to the global decline in their populations worldwide. Amphibians are one of the most threatened and poorly known groups of vertebrates in several geographic areas, even though they play a central role in their own ecosystems. At different levels, amphibians make their contribution to proper ecosystem functioning. They act as regulators of the food web and nutrient cycling, and they also provide several valuable ecosystem services, e.g., as a food source and as animal models for lab research. In this sense, it seems clear that the maintenance of amphibian diversity should be one of the major goals for the several countries where their population decline is observed. However, we are still struggling with the very first step of this process, i.e., the correct identification of the amphibian species diversity. Over the past few decades, research on molecular identification of amphibians using DNA barcoding has encountered some difficulties related to high variability in the mitochondrial genome of amphibians, and a research gap is noticeable in the literature. We herein evaluated both COI and 16S rRNA mitochondrial genes for the molecular identification of frogs and tadpoles in a large fragment of the South American Atlantic Rainforest in Rio de Janeiro, Brazil. Our results suggest that both COI and 16S rRNA are informative markers for the molecular identification of the amphibian specimens with all specimens unambiguously identified at the species level. We also made publicly available 12 new sequences of Atlantic Rainforest amphibian species for the first time, and we discussed some conservation issues related to amphibians within the Atlantic Rainforest domains in the state of Rio de Janeiro, Brazil.

## Introduction

Amphibians are the focus of a recent debate and public attention owing to the global decline in their populations worldwide. The Class Amphibia has an estimated 8,004 species [1] with Brazil home to the greatest known species richness, having about 1080 species, of which 1039 are anurans [2–3]. Of these, 543 inhabit the South American Atlantic Rainforest biome in Brazil [4].

One of the world’s most threatened biomes, the South American Atlantic Rainforest originally spread from northeastern Argentina and Paraguay to northeastern Brazil, nowadays has only about 12% of its original cover [5] and is considered one of the world’s biodiversity hotspots [6]. Among all Brazilian states, about 20% of Rio de Janeiro is covered by Atlantic Rainforest remnants [7]. Located between 20–24° S and 45–41°W, it features a heterogeneous landscape which favors the maintenance of high rates of biodiversity and endemism for several groups [8–9] such as Amphibians [9, 10–13].

In the state of Rio de Janeiro, as well as Brazil as a whole, intense industrial and agroindustrial activities are putting exponentially growing pressure on biodiversity [14]. Oil- and gas-related activities, the expansion of agriculture and livestock areas, the changes caused by urban development, and, surely, the effects of climate change are seriously changing the patterns of land use and deeply influencing biotic communities all around the world [15–17].

Among these communities, amphibians are one of the most threatened and poorly known groups of vertebrates in several geographic areas [18–19], even though they play a central role in their own ecosystems. At different levels, amphibians make their contribution to proper ecosystem functioning. They act as regulators of the food web and nutrient cycling [20–21], and they also provide several valuable ecosystem services, e.g., as a food source and as animal models for lab research [21–23]. In this sense, it seems clear that the maintenance of amphibian diversity should be one of the major goals for the several countries where their population decline is observed. However, we are still struggling with the very first step of this process, i.e., the correct identification of the amphibian species diversity.

Most amphibian species have a complex life cycle, with aquatic larval and terrestrial adult stages, being susceptible to disturbances in both environments [24–26]. Therefore, amphibians are commonly used as a biological indicator of habitat degradation and environmental health status in both aquatic and terrestrial habitats [18,27–31].

Several studies on anuran have explored the variation and characteristics of the postmetamorphic stages, with tadpole morphological characters and ecology comparatively less studied [32–33]. However, the tadpole is the stage most frequently found and easiest registered in nature for several anuran species, by remaining in the breeding spot for longer periods than adults [34–36]. Although the number of studies on tadpoles has increased [33,37–38], the tadpoles of the majority of tropical anuran species remain unknown [39]. Amphibians have been declining from the planet at an alarming rate, and related to the spread of pathogens into wild (including in protected areas) or due to unknown causes [18,33,40–42]. In this sense, conservation and management actions regarding anurans must involve information on the different life stages to ensure efficient and comprehensive conservation plans for the species [33].

A major drawback for the use of tadpole data in conservation programs is the difficulties of appropriated species identification in such early developmental stages. Molecular methods offer a straightforward alternative for the identification of several organisms such as anurans, including its eggs and larval stages. The DNA barcoding methodology uses a short, standardized gene fragment for species identification [43–44] being a rapid and low-cost method [45]. This method has been already used in the identification of many taxa, such as amphibians [31,42,46], birds [47–48], bivalves [49], butterflies [50–51], spiders [52], fishes [53–54], and mammals [55]. In the case of amphibians, a useful application of DNA barcoding is the identification of larvae of some species, especially of those species whose tadpoles are considerably distinct morphologically from their adult phase [31,35,42]. However, sequence variation at the COI priming sites leads to problems in the use of universal primers in amphibian barcoding [31,42,56]. A mixture of several primers is needed to reliably amplify the COI gene from all amphibian species. The use of alternative markers with more conserved priming sites, such as 16S rRNA is often used for identifying amphibian species [31,57]. This calls for the collection of field data and species inventories to guide management and conservation actions and policies all around the world, including Brazil [58]. However, despite the increasing number of amphibian and/or reptile studies for various localities in the South American Atlantic Rainforest, species lists for various localities are still needed in order to provide a comprehensive characterization of their herpetofaunas [59].

Here, we present a comprehensive identification of adult frogs and tadpoles in the REGUA area. Aimed at precise identification of REGUA amphibian fauna, we lay the groundwork for proper management strategies for this area and the remaining Atlantic Forest fragments. To ensure this precision, we have used DNA barcoding with partial COI and 16S rRNA sequences of adult frogs and tadpoles.

## Material and methods

**Fig 1:**
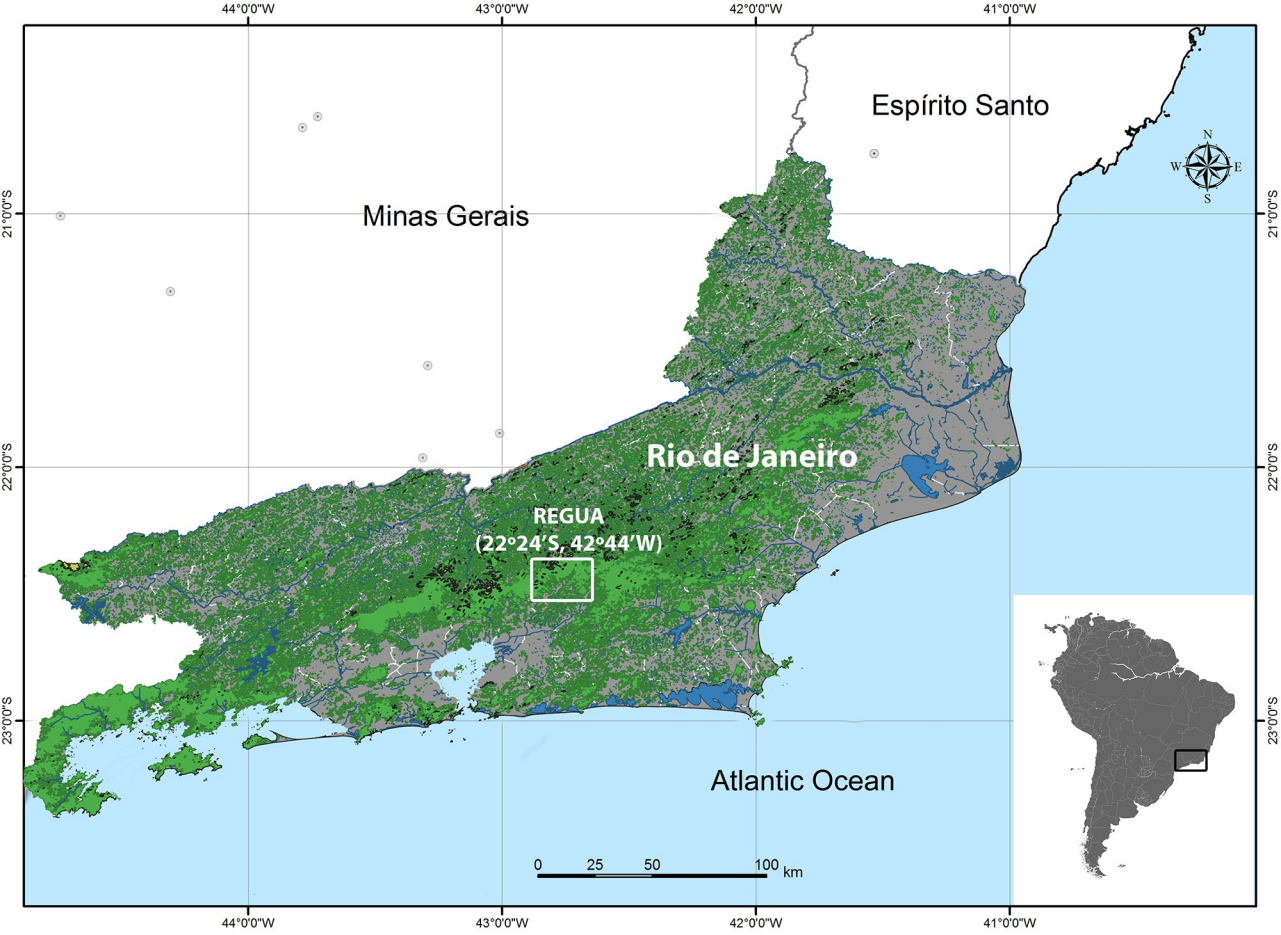
Study site. Map of Rio de Janeiro State, Brazil, showing the Reserva Ecológica de Guapiaçú (REGUA) from which specimens were sampled. Atlantic Rainforest remaining fragments are represented in dark and light green; continental waters in dark blue; urbanized area in gray. The map was made using the QGIS software with shapefiles from the IBGE Database (www.ibge.gov.br).

Tissue samples from 88 specimens, including adults (n = 50 specimens from 23 species) and tadpoles (n = 38 specimens from 12 species), were collected in an Atlantic Rainforest fragment in Cachoeiras de Macacu (REGUA), Rio de Janeiro, Brazil (Fig 1). A complete list of all species grouped into its eight distinct families is presented in Table 1. The Reserva Ecológica de Guapiaçu (REGUA—22°24’S, 42°44’W) comprises an area of about 7,600 ha. REGUA is part of a much larger continuous and protected forest area in Rio de Janeiro, Brazil. Together with other conservation units, such as the Parque Estadual dos Três Picos (ca. 46,000 ha), Parque Nacional da Serra dos Órgãos (ca.11,800 ha), and Estação Ecológica Estadual do Paraíso (ca.5,000 ha), REGUA is part of a large contiguous protected area of wet Atlantic Forest, mostly represented by montane and low montane rainforest.

**Table 1:** List of collected species from REGUA area.

Family	Species	Number of individuals	Tadpoles	Adults
**Bufonidae**				
	*Rhinella ornata*	7	5	2
**Cycloramphidae**				
	*Cycloramphus brasiliensis*	1	-	1
	*Thoropa miliaris*	1	-	1
**Hylidae**				
	*Aplastodiscus arildae*	5	4	1
	*Dendropsophus berthalutzae*	2	-	2
	*Dendropsophus bipunctatus*	2	-	2
	*Dendropsophus meridianus*	3	-	3
	*Hypsiboas secedens*	1	-	1
	*Hypsiboas semilineatus*	6	3	3
	*Scinax albicans*	8	4	4
	*Scinax flavoguttatus*	5	3	2
	*Scinax humilis*	1	-	1
**Hylodidae**				
	*Crossodactylus aeneus*	13	7	6
	*Hylodes asper*	1	1	-
	*Hylodes charadranaetes*	8	2	6
	*Hylodes lateristrigatus*	7	5	2
	*Hylodes pipilans*	2	1	1
**Leiuperidae**				
	*Physalaemus signifer*	2	-	2
**Leptodactylidae**				
	*Adenomera marmoratus*	3	-	3
	*Megaelosia goeldii*	1	-	1
**Odontophrynidae**				
	*Proceratophrys appendiculata*	3	1	2
	*Proceratophrys boiei*	4	3	1
**Phyllomedusidae**				
	*Phyllomedusa burmeisteri*	3	-	3
	**Total individuals**	**88**	**38**	**50**

**Fig 2:**
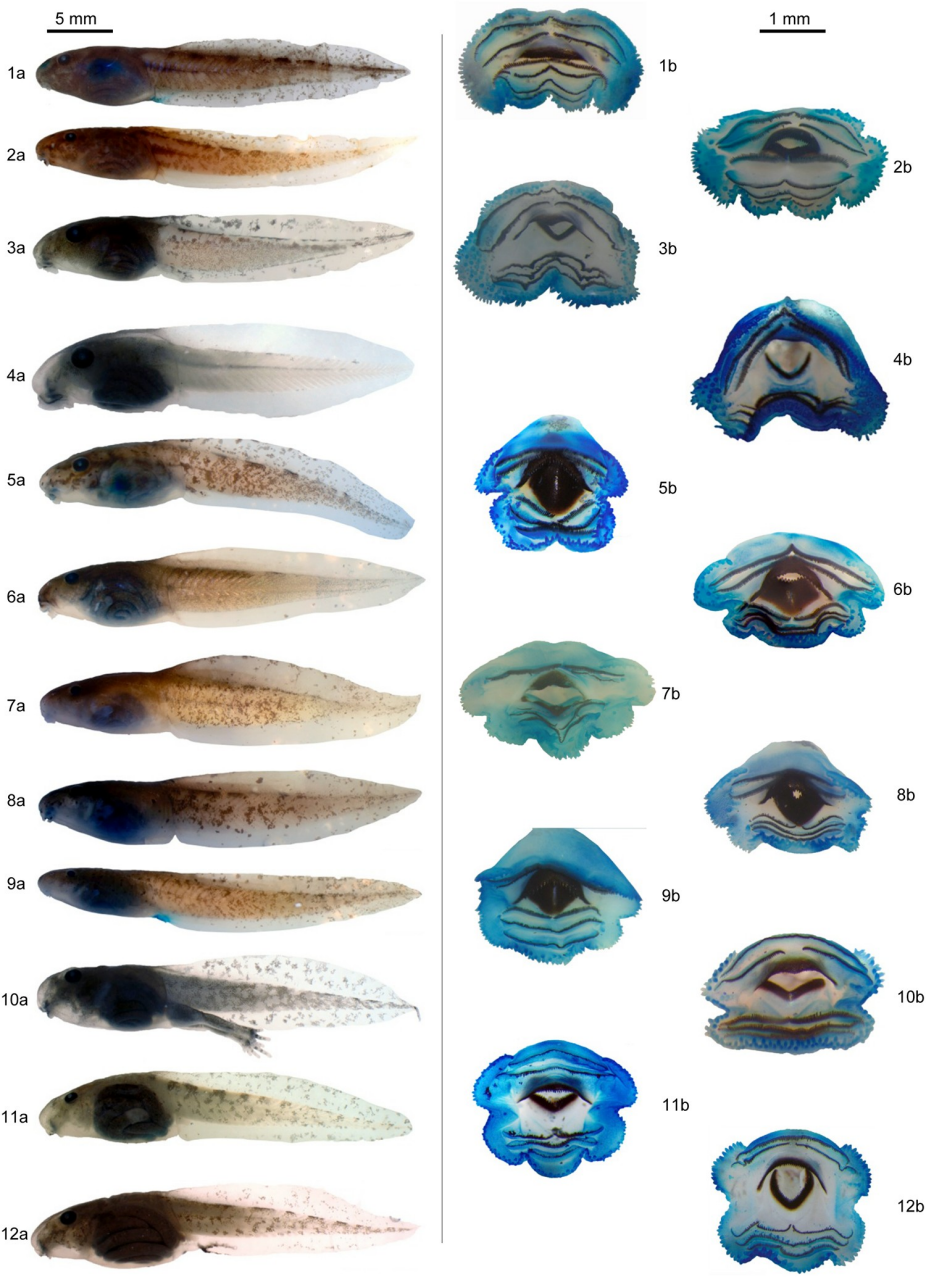
Tadpoles. Lateral view (a) and oral disk (b) of 1. *Aplastodiscus arildae*; 2. *Bokermannohyla circumdata*; 3. *Scinax albicans*; 4. *Scinax flavoguttatus*; 5. *Crossodactylus aeneus*; 6. *Hylodes asper*; 7. *Hylodes charadranaetes*; 8. *Hylodes lateristrigatus*; 9. *Hylodes pipilans*; 10. *Physalaemus signifer*; 11. *Proceratophrys appendiculata*; and 12. *Proceratophrys boiei*.

The adults were morphologically identified and classified into 23 species. The tadpoles were cleared and stained for morphological observation and classification (Fig 2). All tissue samples (muscle) were extracted from dead specimens collected with permission under the approved authorization number 18684 issued by ICMBio (Instituto Chico Mendes de Conservação da Biodiversidade). All techniques used to capture, sample, and euthanize were performed to minimize animal suffering and followed the guidelines provided by the Herpetological Animal Care and Use Committee (HACC) of the American Society of Ichthyologists and Herpetologists. The collected individuals were euthanized with an anesthetic application (5% Lidocaine) over the skin. The voucher specimens were fixed in formalin and deposited on the reference collection of the Ecology Department. The samples were preserved in alcohol 70%.

For molecular identification, small fragments of liver and leg muscle were removed from adult amphibians. In the case of tadpoles, fragments of tail and muscle were collected. All samples were submitted to the standard protocol for DNA extraction and purification from the Qiagen QIAmp^®^ DNA FFPE Tissue Kit. Amplification and sequencing of 16S rDNA gene fragments used the following primers: 16SrA-L (5’-CGC CTG TTT ATC AAA AAC AT-3’) and 16SrB-H (5’ -CCG GTC TGA ACT CAG ATC ACG T-3’) [60]. PCR amplifications were performed in a 20 μL volume reaction containing 1 μL (2 ng) of genomic DNA, 12 μL of ddH_2_O, 0.5 μM of 4X Platinum buffer (Thermo Fisher), 10 μM of each primer and 0.2 μL (1U) of Taq Platinum. The PCR program consisted of 2 minutes at 94°C, 35 cycles of 30 seconds at 94°, 40 seconds at 52°C, and 1 minute at 72°C, with a final extension step for 10 minutes at 72°C. The COI 5’ end gene region was amplified using the following primers: AnF1 (5’-HAA YCA YAA AGA YAT YGG-3’) and AnR1 (5’-CCR AAR AAT CAR AAD ARR TGT TG-3’) [61] Frog samples for *Adenomera marmoratus*, *Phyllomedusa burmeisteri* and *Thoropa miliaris* were provided by donation by M. Lyra from the Universidade Estadual Paulista (UNESP) and individual information about the specimens could be checked on [61]. PCR amplifications were performed in a 25μL volume reaction containing 2 μL (4 ng) of genomic DNA, 16.5 μL of ddH_2_O, 0.2 μM of 4X Platinum buffer (Thermo Fisher), 10 μM of each primer and 0.2 μL (1U) of Taq Platinum. The PCR profiles consisted of 3 minutes at 95°C, 5 cycles of 30 seconds at 95°, 30 seconds at 48°C, and 1 minute at 60°C; 30 cycles of 30 seconds at 95°, 30 seconds at 50°C, and 1 minute at 60°C with a final extension step for 5 minutes at 60°C, as previously described for the 16S. The sequencing of the COI gene used M13 extensions tails M13F (5’-TGT AAA ACG ACG GCC AGT-3’) and M13R (5’CAG GAA ACA GCT ATG AC-3’) to maximize the sequencing reliability on the first bases of the COI gene.

The sequencing reactions were performed with BigDye^®^ Terminator v3.1 Cycle Sequencing kit (Thermo Fisher) with 10 seconds at 95°C, 25 cycles of 5 minutes at 50°C, and 4 minutes at 60°C. Sequencing products were processed in an ABI 3500 capillary system (Thermo Fisher). To ensure the absence of saturation in both datasets, both databases (COI and 16S rRNA) were evaluated with and without the 3^rd^ codon position. The neighbor-joining (NJ) and maximum-likelihood (ML) trees were constructed using MEGA 7.0 software [62]. The NJ sequence divergences were calculated based on the Kimura 2-parameter (K2P) distance model with bootstrap support analysis (1,000 replicates), while the maximum-likelihood (ML) result was also generated with bootstrap support analysis (1,000 replicates) with the GTR+G+I nucleotide substitution model chosen as the best fit model based on the AKAYKE (AIC) criterion.

## Results

The 50 adult frogs sampled for this study represent 23 distinct species, which is about 31% of the 73 amphibian species registered for the area of REGUA [59]. Tadpoles were represented by 38 individuals. The degenerate PCR primers successfully amplified and sequenced the COI gene region for all 88 analyzed specimens, suggesting that it is a uniquely useful primer pair for the molecular identification of amphibians. No saturation signals on the constructed datasets were observed after evaluation of resultant topologies with and without the 3^rd^ codon position. GenBank accession numbers of the retrieved COI and 16S rRNA reference sequences are available in Table 2. Field and voucher data are available in the S1 File.

**Table 2:** GenBank accession numbers for reference sequences used in the present manuscript.

Species	16S	COI
	GenBank	GenBank
*Rhinella ornata*	KM390776	KU234691
*Cycloramphus brasiliensis*	KM390777	KU234692
*Proceratophrys appendiculata*	KM390778	KU234693
*Proceratophrys boiei*	KM390779	KU234694
*Thoropa miliaris*	KM390780	KU234695
*Aplastodicus arildae*	KM390781	KU234696
*Dendropsophus berthalutzae*	KM390782	KU234697
*Dendropsophus bipunctatus*	KM390783	KU234698
*Dendropsophus meridianus*	KM390784	KU234699
*Hypsiboas secedens*	KM390785	KU234700
*Hypsiboas semilineatus*	KM390786	KU234701
*Phyllomedusa burmeisteri*	KM390787	KU234702
*Scinax albicans*	KM390788	KU234703
*Scinax flavoguttatus*	KM390789	KU234704
*Scinax humilis*	KM390790	KU234705
*Corssodactylus aeneus*	KM390791	KU234706
*Hylodes asper*	KM390792	KU234707
*Hylodes charadranaetes*	KM390793	KU234708
*Hylodes lateristrigatus*	KM390794	KU234709
*Hylodes pipilans*	KM390795	KU234710
*Megaelosia goeldii*	KM390796	KU234711
*Physalaemus signifier*	KM390797	KU234712
*Adenomera marmoratus*	KM390798	KU234713

### New publicly available amphibian sequences

Among all the retrieved sequences of the 23 species found within the REGUA area in the present manuscript, 12 represent new sequences from species that were, for the first time, deposited and made publicly available. They are the sequences for the following species: *Crossodactylus aeneus* (KU234706), *Cycloramphus brasiliensis* (KU234692), *Dendropsophus bipunctatus* (KU234698), *Dendropsophus meridianus* (KU234699), *Hylodes charadranaetes* (KU234708), *Hylodes lateristrigatus* (KU234709), *Hypsiboas secedens* (KU234700), *Phyllomedusa burmeisteri* (KU234702), *Physalaemus signifier* (KU234712), *Scinax albicans* (KU234703), *Scinax flavoguttatus* (KU234704), and *Scinax humilis* (KU234705). The sequences for *Hylodes pipilans* (KU234710) and *Megaelosia goeldii* (KU234711) are only the second available molecular record for these two species. The databases, including all sequences used in the present manuscript, are available in the S2 File.

### Molecular identification

The collected data corresponds to eight amphibian families, as follows: Bufonidae, Cycloramphidae, Hylidae, Hylodidae, Leiuperidae, Leptodactylidae, Odontophrynidae, and Phyllomedusidae. All exhibit both adult and tadpole specimens except for the Cycloramphidae, the Leiuperidae, and the Leptodactylidae. All Neighbor-Joining (K2P) trees can be seen in the S3 File. All divergence values in this section were based on the COI K2P distances, and the datasets used for distance calculations are also available upon request. Only nodes presenting over 50% of bootstrap support values were presented and discussed in the following text.

The Bufonidae was represented by one unique species, *Rhinella ornata*. The obtained sequences were grouped with previously available sequences of *R*. *ornata* and *R*. *crucifer* in a clade exhibiting 0.8% of mean distance and with 9.3% divergence of the closest neighbor, *R*. *icterica*.

The Cycloramphidae was represented by two species, *Cycloramphus brasiliensis* and *Thoropa miliaris*. *C*. *brasiliensis* was clearly recovered as a monophyletic clade and diverged 20.2% from its closest neighbor, *C*. *bandeirensis*. *Thoropa miliaris* was also recovered and grouped as a monophyletic clade although it exhibits 8.3% of divergence within the species and 16.9% of divergence from its closest neighbor, *T*. *taophora*.

The Hylidae was by far the most represented family within our dataset. It was represented by 10 species, including *Aplastodiscus arildae*, *Dendropsophus berthalutzae*, *D*. *bipunctatus*, *D*. *meridianus*, *Hypsiboas secedens*, *H*. *semilineatus*, *Scinax albicans*, *S*. *flavoguttatus*, and *S*. *humilis*. Initially regarded as *Aplastodiscus eugenioi* based on morphological identification, the REGUA specimens of *Aplastodiscus* were recovered and grouped with *A*. *arildae*, exhibiting 12.4% of divergence from previously published sequences of *A*. *arildae*. The closest neighbors were *A*. *albofrenatus* with 12.9% of divergence and *A*. *eugenioi*, which exhibited 13.9% of divergence. The genus *Dendropsophus* was represented by three species. *D*. *berthalutzae* grouped with the unique sequence publicly available for the species although with a divergence of 18.9%. The remaining two species were *D*. *meridianus* and *D*. *bipunctatus*, both newly available sequences for the species. *D*. *meridianus* was recovered in a node together with *D*. *vraemi* in half of the analyses, presenting a divergence of 20.2%, while *D*. *bipunctatus* was recovered in a node with low bootstrap support value. The genus *Hypsiboas* was represented by two species, *H*. *secedens* and *H*. *semilineatus*. The obtained sequence for *H*. *secedens* was the first one available for this species. It was also recovered in a low support node; however, *H*. *semilineatus* was clearly recovered together with previously available sequences of the same species with 5.3% of divergence The group formed by both collected and previously available sequences of *H*. *semilineatus* was recovered in a group with the species *H*. *boans*, diverging 13.5%, and *H*. *wavrini*, which presented 13.4% of divergence. The last Hylidae genus in our dataset, *Scinax*, was represented by three species, including *Scinax albicans*, *S*. *flavoguttatus*, and *S*. *humilis*. Although recovered clearly grouped, both *S*. *flavoguttatus* and *S*. *humilis* are newly sequenced species, and reference sequences are not available for comparison. *Scinax albicans* was recovered as monophyletic and grouped to its closest neighbor, *S*. *obtriangulatus*, exhibiting a distance of 17.3%.

The Hylodidae was represented by five species grouped into two genera, *Crossodactylus* and *Hylodes*. *Crossodactylus aeneus* sequences are new, and the unique available sequences for comparison came from *C*. *caramaschii*, which diverged from *C*. *aeneus* by 19.1%. *C*. *aeneus* also exhibited within divergence of 3.7%. The genus *Hylodes* was respresented by four species, including *Hylodes asper* and *H*. *pipilans* with available sequences for comparison and the new *H*. *charadranaetes* and *H*. *lateristrigatus*. *Hylodes asper* grouped with available *H*. *asper* sequences although they exhibited 9.2% of divergence from them, while *H*. *pipilans* also grouped with available *H*. *pipilans* sequences, but presented only 6.2% of intraspecific divergence.

The Leiuperidae was represented uniquely by *Physalaemus signifier* which was recovered in a group that includes the basal *P*. *nattereri*, and, sequentially, *P*. *signifier*, *P*. *bokermanni*, *P*. *atlanticus*, and *P*. *moreirae*. *P*. *signifier* exhibited divergence of 15.5% from *P*. *bokermanni*, its closest neighbor within the group.

The Leptodactylidae was represented by two species, *Adenomera marmorata* and *Megaelosia goeldii*. *Adenomera marmorata* was recovered and grouped with available sequences of *A*. *marmorata* and presented *A*. *ajurauna* as the closest neighbor, exhibiting 13.3% of divergence from it and 1.3% of intraspecific divergence, while *A*. *ajurauna* exhibited 5.9% of divergence within the species. *Megaelosia goeldi* was also recovered and grouped with previously available *M*. *goeldi* sequences although it exhibited divergence of 7.2% from the unique previously published sequence.

The family Odontophrynidae was represented by *Proceratophrys boiei* and *P*. *appendiculata*. Both species were successfully recovered and grouped with previously available sequences for the species, diverging 7.1% and 2.3% from them, respectively.

The unique Phyllomedusidae *Phyllomedusa burmeisteri* is newly sequenced, and no previous sequences are available for comparison. It was recovered together with *P*. *distincta*, exhibiting 8.9% of divergence.

Finally, within distances among the several species herein surveyed ranged from quite small distances, such as 0.1% found within *Scinax albicans* sequences, to 3.2% − 3.7% found for *Proceratophrys boiei* and *Crossodactylus aeneus* and 7.5% − 8.3% found within *Aplastodiscus arildae* and *Thoropa miliaris*, respectively.

### Tadpole identification

**Fig 3:**
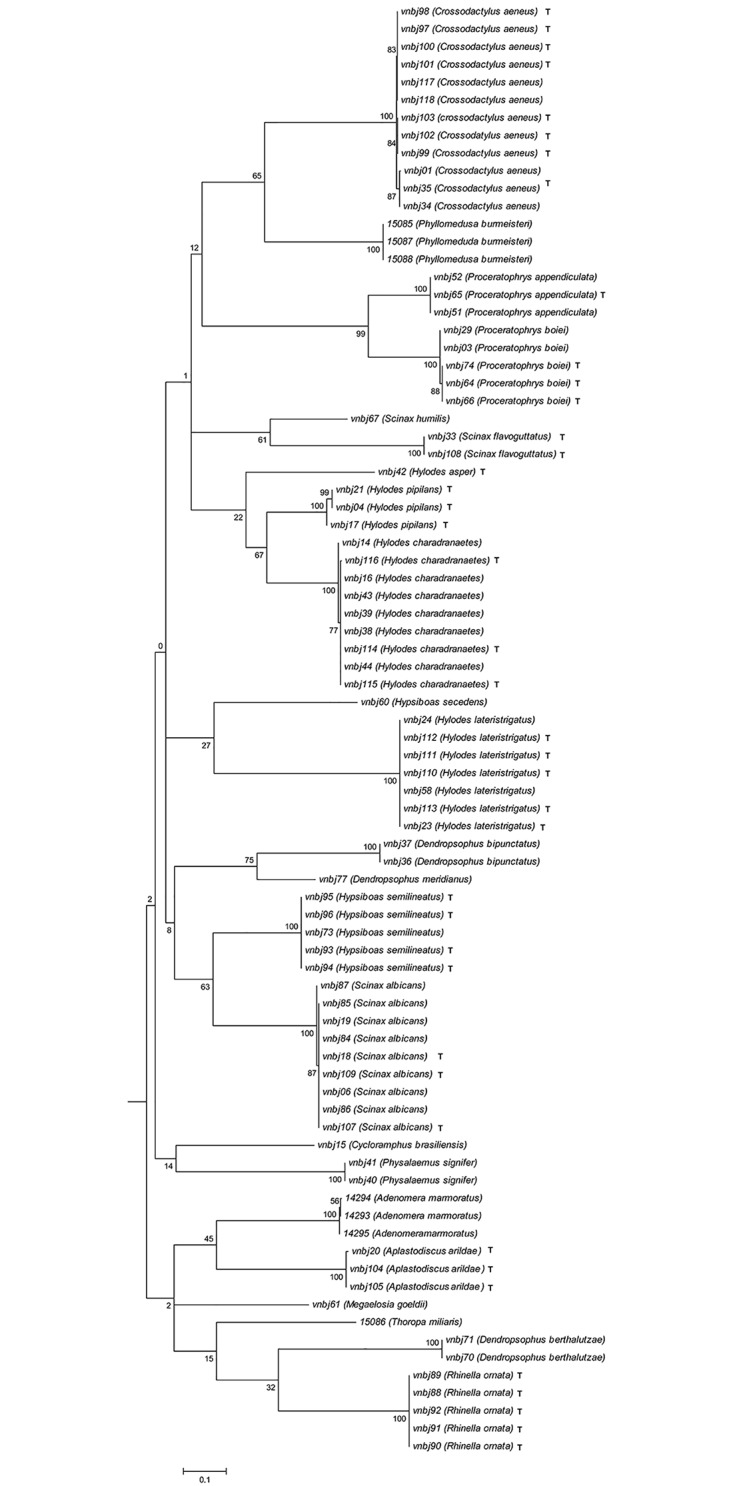
Neighbor-joining (K2P) tree of the COI gene. Bootstrap probabilities near each internal node. Tadpoles are marked with a T after the species epithet. All remaining specimens comprise adults from the reference database.

**Fig 4:**
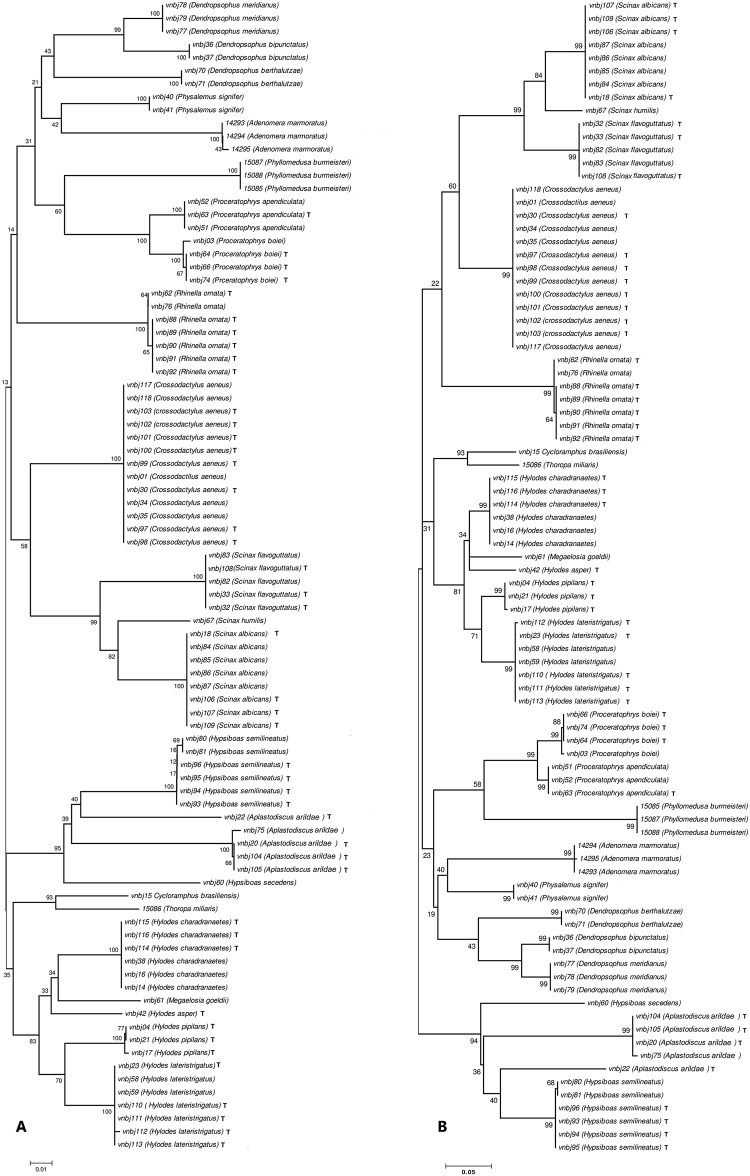
Neighbor-joining (A) and maximum-likelihood (B) trees of the 16S rRNA gene. Bootstrap probabilities near each internal node. Tadpoles are marked with a T after the species epithet. All remaining specimens comprise adults from the reference database.

The topologies observed for both COI and 16S rRNA were comparable and supported by high bootstrap values. All tadpoles were unambiguously identified at species level based on the reference database built with the adult frogs. The COI and 16S NJ trees, including both tadpoles and specimens from the reference database (adult frogs), show that all species were recovered as monophyletic groups with high bootstrap support (Figs 3 and 4). The overall COI K2P mean distance is 0.245, ranging from 0.187 between *Proceratophrys boiei* and *Proceratophrys appendiculata* to 0.340 between *Scinax flavoguttatus* and *Rhinella ornata*.

As an alternative molecular marker, 16S rRNA barcoding correctly identified all species in the present manuscript. The neighbor-joining (NJ) and maximum-likelihood (ML) resultant trees were presented in Fig 4A and 4B, and they show that all individuals were recovered as monophyletic group with high bootstrap support. For both NJ and ML trees, specimen *vnbj22 Aplatodiscus arildae* was recovered outside of the group formed by the remaining individuals of the same species. The 16S rRNA interspecific distances were also calculated for all species. The values ranged from 0.039 between *Proceratophrys boiei* and *P*. *appendiculata* to 0.189 between *Hylodes asper* and *Aplastodiscus arildae*.

A complete table including COI and 16S rRNA K2P distances among collected species is available in both S4 and S5 Files.

## Discussion and conclusions

The state of Rio de Janeiro is home to about 40% of the South American Atlantic Rainforest amphibian species. About 19% is regarded as data deficient, meaning that the conservation status of these species has not yet been evaluated and that the number of threatened species within the South American Atlantic Rainforest could be higher.

Together with the anthropogenic pressures related to habitat fragmentation and destruction, the presence of exotic invasive species also threatens survivability of amphibian fauna within the South American Atlantic Rainforest. Van Sluys et al. [63] reported the expansion of the exotic American bullfrog (*Lithobates catesbeianus*) within the state of Rio de Janeiro, pointing to the need for population monitoring and management programs for removal of individuals from natural habitats and, thereby, favoring the maintenance of native species and populations.

Recently, [13] presented the last checklist of amphibian species from the Atlantic Rainforest in Rio de Janeiro, indicating that the amphibian richness presented is still underestimated, tending to increase in the following years as a result of new taxonomic studies and local herpetofaunal surveys. The authors also pointed out the importance of Rio de Janeiro state as a key spot for amphibian diversity, not only for the Atlantic Rainforest, but for Brazilian amphibian fauna as a whole.

Amphibians are commonly regarded as a challenging group for molecular identification approaches and the usefulness of these methods within amphibians has already been discussed in several manuscripts over the last decades [31,64–66]. Difficulties related to the high variability of their mitochondrial genome and the genetic structure of their recognized species make identification of amphibian species based on the well- accepted DNA barcoding methodology a kind of outlier among herpetologists. The DNA barcoding sequence, or the first 650bp of the Cytochrome C oxidase subunit I gene, as proposed by [43], is highly polymorphic among amphibians, and its use for species identification depends on a mixture of various primers to correctly amplify all species, mainly from the high level of polymorphisms observed on the common COI priming sites. Difficulties could be also observed when assessing the population level of amphibian diversity where the analysis and comparison of mitochondrial and nuclear datasets is rarely concordant, and events of occasional hybridization and introgression commonly affect amphibian species and their population substructure. In this sense, it is clear that species delimitation among amphibians does not seem to be an easy task, especially using only the COI barcoding approach. It is well accepted that the use of alternative markers, such as the more preserved 16S rRNA, should be taken into account for amphibians, the species of which are relatively old entities and exhibit a high amount of mutations [66].

Our results for both COI and 16S rRNA (Figs 3 and 4) showed that at least within the limits of the obtained databases, all species were clearly recovered as monophyletic units, therefore making the DNA barcoding approach herein used a successful methodology for species diversity assessment within the analyzed fragment of the South American Atlantic Rainforest. As expected, the interspecific divergence was consistent, ranging from 0.187 to 0.340, while the intraspecific divergence was tiny for almost all the species, ranging from 0.002 to 0.007, except for *Hylodes charadranaetes*, which presented an intraspecific divergence of 0.042. However, independent of the result observed for *H*. *charadranaetes*, the divergence observed was sufficient to ensure a useful barcoding gap among all the analyzed species.

The recent study of [23] also reached high levels of success using COI barcoding on the molecular identification of amphibian species from west Central Africa, as well as those already obtained by [46,61].

Vences et al. [31] also pointed out the tadpole / adult correlation as one of the major goals for using molecular identification methodologies. The similarity observed for several species included in the present survey (Fig 2) is remarkable, even with stained sections, such as the buccal apparatus, a morphological complex commonly used for species determination on tadpoles. The identification of tadpoles is surely one of the major challenges in studying amphibians by virtue of being the most fragile life stage of anurans [67–68]. The patterns of convergence and parallelism among relatively closely related taxa constitute a particular challenge to the study of tadpoles in the field [69]. The tadpoles of many anuran species have not been morphologically described [39,70]. Furthermore, tadpoles of related species can be externally similar in morphology. In such cases, genetic analyses have been considered a successful tool to identify tadpoles at the species level [31,70]. This success was surely reached herein since all the tadpoles were correctly assigned to their species. Tadpole molecular monitoring is a positive step for determining the amphibian species richness of a given area. They are the most common and easily found form for several amphibian species within the South American Atlantic Rainforest. They are the most persistent stage, staying in the same area/pond for several days and probably weeks, making them an easy target for sampling.

Finally, we do believe, as already observed by [46,61], that the DNA barcoding methodology could be clearly used for amphibian species identification and richness estimation, therefore guiding the development of appropriate conservation and management strategies. We also agree, as already mentioned by [46], that it is far more advantageous to include amphibians in a DNA barcoding initiative rather than excluding them. The BOLD database is a huge achievement for several zoological groups all around the world, but amphibians were left by the wayside in recent years because of a supposed inability to use the technology for species identification. However, it seems obvious that correct taxonomic identification depends on the comprehensiveness of the dataset built for comparative purposes, and this concern is surely not restricted to amphibians. In this sense and regarding all threats amphibians are facing nowadays, the construction of reliable databases is urgent, and we should focus on the construction of publicly available datasets of reference sequences that include geographical and ecological information, as well as any other kind of data that support proper species identification, in the effort to support ecologists and conservation biologists as they struggle to develop strategies of amphibian fauna management and conservation within the South American Atlantic Rainforest.

## Supporting information

S1 File Field data and voucher numbers. (XLSX)

S2 File Databases used in the present manuscript. (ZIP)

S3 File NJ trees. NJ K2P trees for each studied taxon.
(PDF)

S4 File COI distances. COI K2P distances for the analyzed groups.
(TIF)

S5 File 16S rRNA distances. 16s rRNA distances for the analyzed groups.
(PDF)
