# Correction: Amphibians on the hotspot: Molecular biology and conservation in the South American Atlantic Rainforest

**DOI:** 10.1371/journal.pone.0227329

**Published:** 2020-01-15

**Authors:** Cesar R. L. Amaral, Anna C. S. Chaves, Vitor N. T. Borges Júnior, Filipe Pereira, Bruna M. Silva, Dayse A. Silva, António Amorim, Elizeu F. Carvalho, Carlos F. D. Rocha

The following information is missing from the Funding statement: This project was supported by the CAPES-PrInt program.
