# Supplementary material for: Amphibians on the hotspot: Molecular biology and conservation in the South American Atlantic Rainforest
Source: PLoS One. 2019 Oct 23;14(10):e0224320. doi: 10.1371/journal.pone.0224320 (PMC6808428; doi:10.1371/journal.pone.0224320)
Supplement: S3 File — NJ K2P trees for each studied taxon. (PDF) [file pone.0224320.s003.pdf]

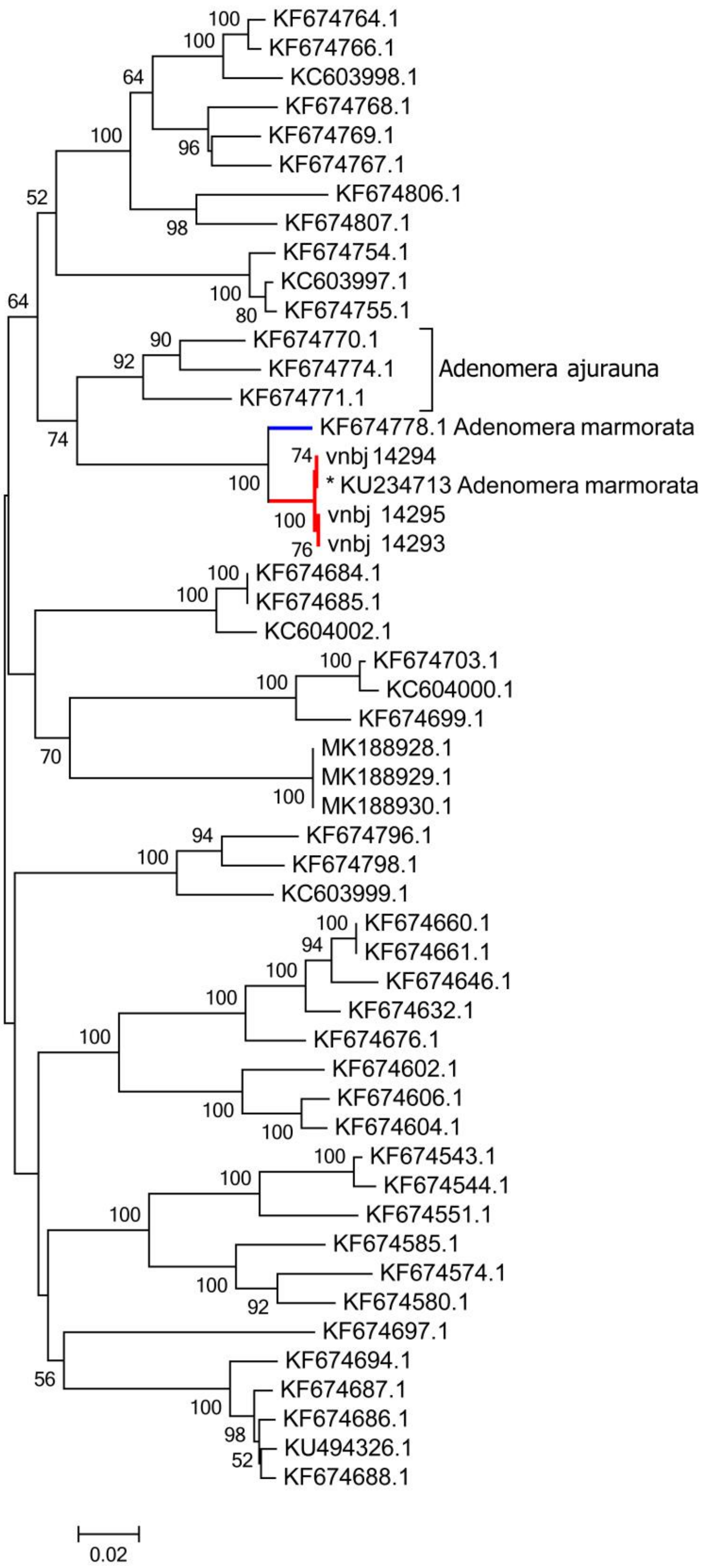

Figure 1 – Neighbor-Joining tree based on the K2P distance of *Adenomera marmorata* from the Reserva Ecológica de Guapiaçu, Rio de Janeiro, Brazil, and close relatives. Bootstrap support values over 50% exhibited close to each node. Branches of collected adults and tadpoles specimens in red. Branches for close related specimens of the same species marked in blue.

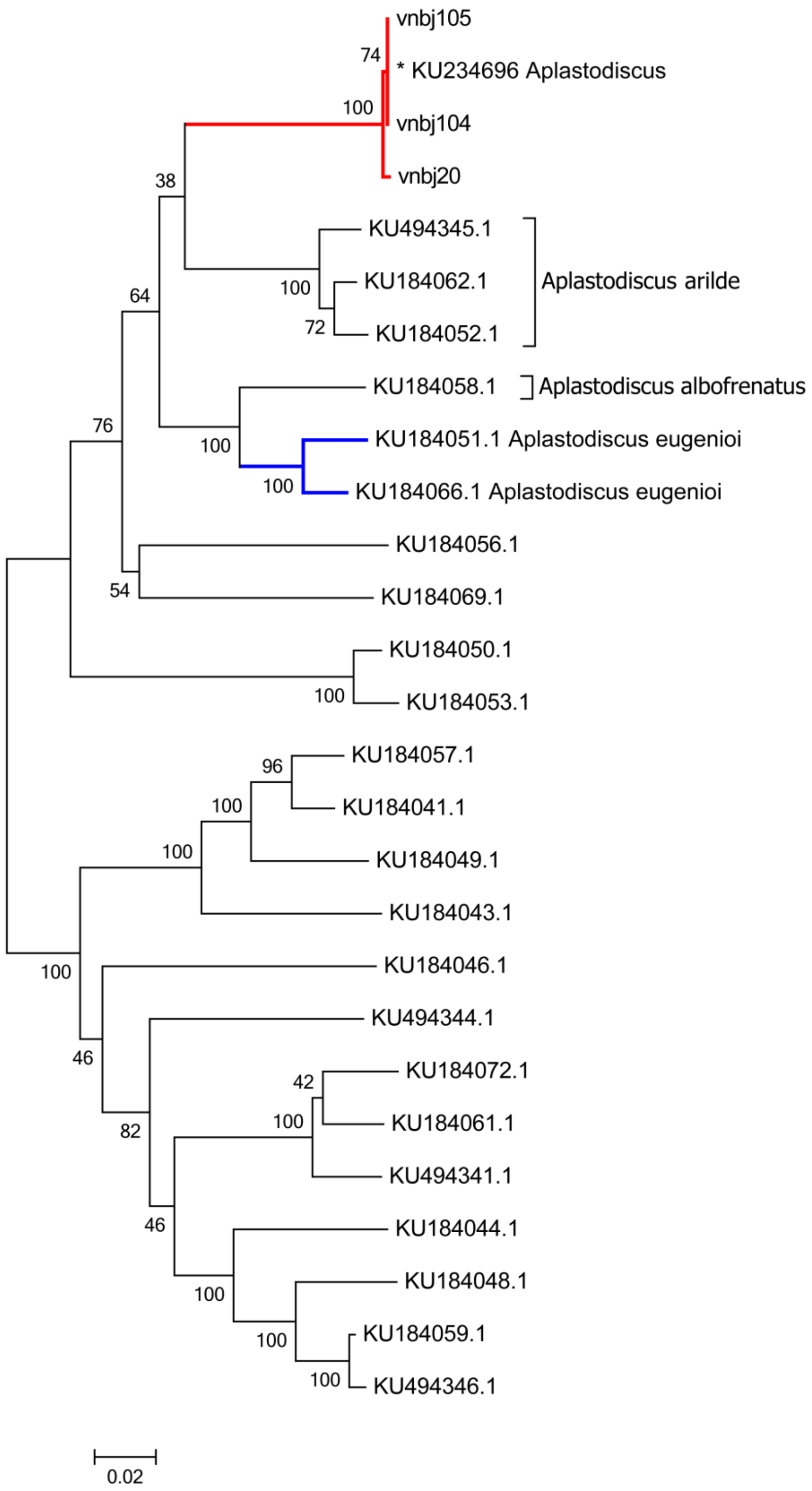

Figure 2 – Neighbor-Joining tree based on the K2P distance of *Aplastodiscus sp.* from the Reserva Ecológica de Guapiaçu, Rio de Janeiro, Brazil, and close relatives. Bootstrap support values over 50% exhibited close to each node. Branches of collected adults and tadpoles specimens in red. Branches for close related specimens of the same species marked in blue.

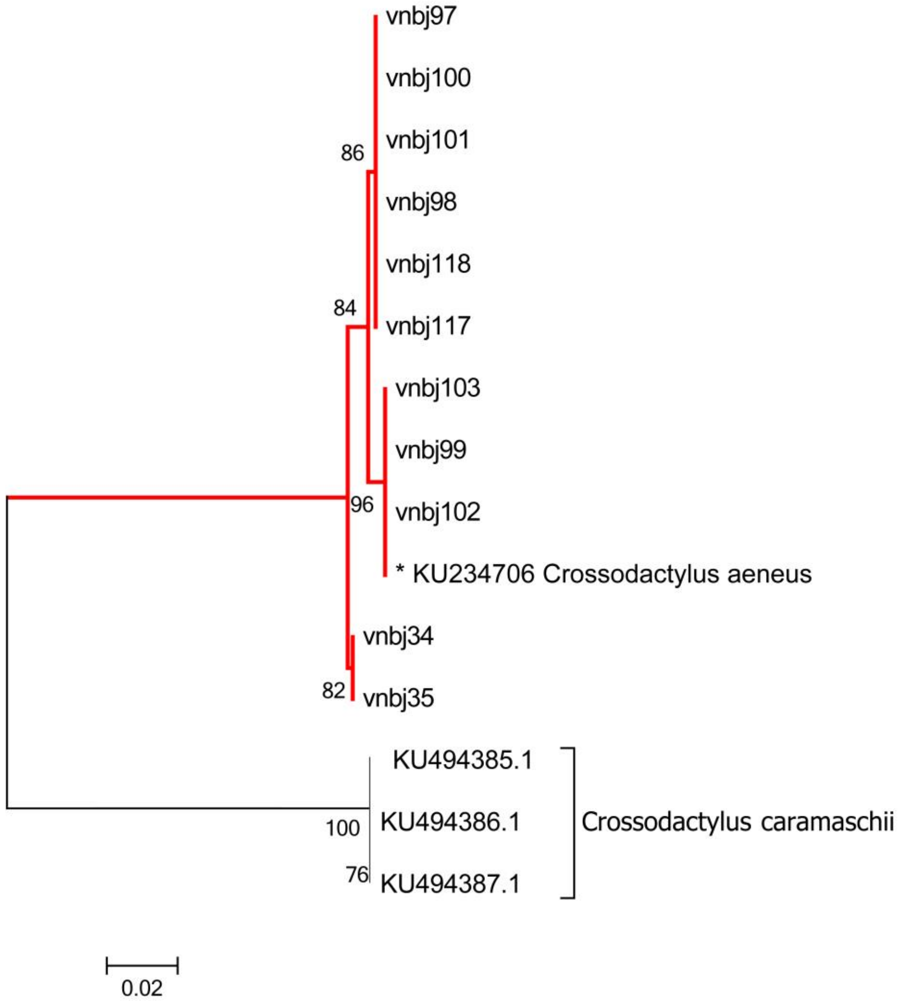

Figure 3 – Neighbor-Joining tree based on the K2P distance of *Crossodactylus aeneus* from the Reserva Ecológica de Guapiaçu, Rio de Janeiro, Brazil, and close relatives. Bootstrap support values over 50% exhibited close to each node. Branches of collected adults and tadpoles specimens in red.

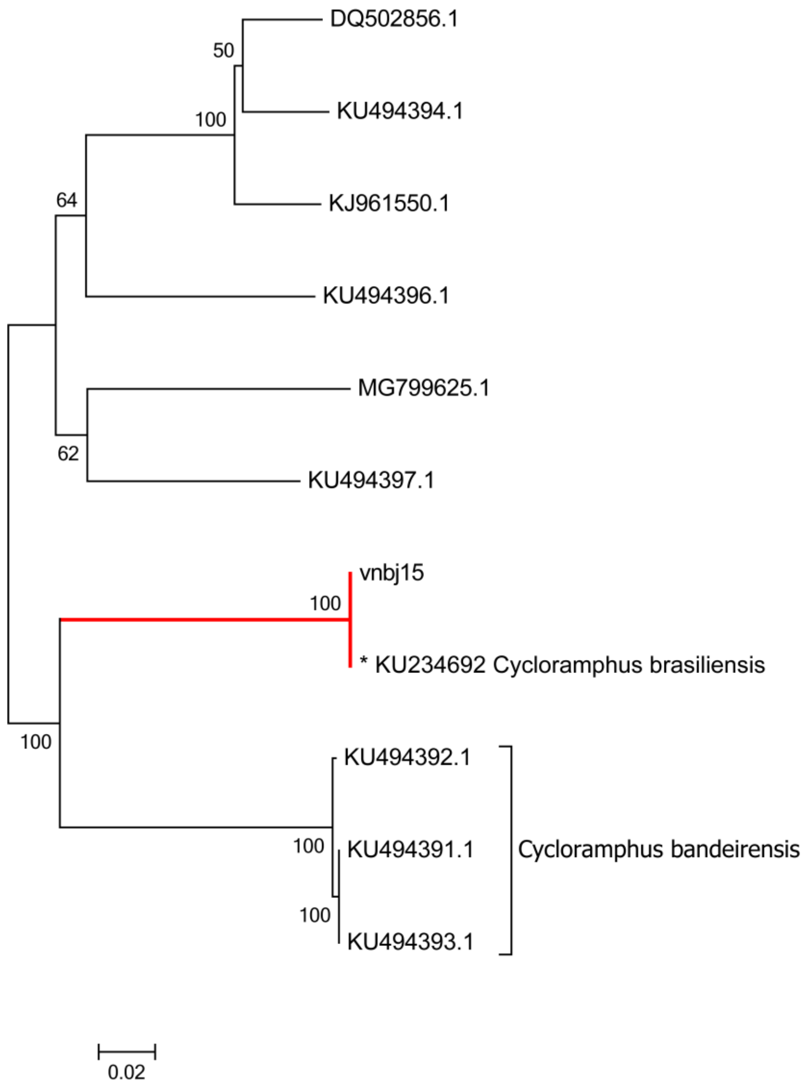

Figure 4 – Neighbor-Joining tree based on the K2P distance of *Cycloramphus brasiliensis* from the Reserva Ecológica de Guapiaçu, Rio de Janeiro, Brazil, and close relatives. Bootstrap support values over 50% exhibited close to each node. Branches of collected adults and tadpoles specimens in red.

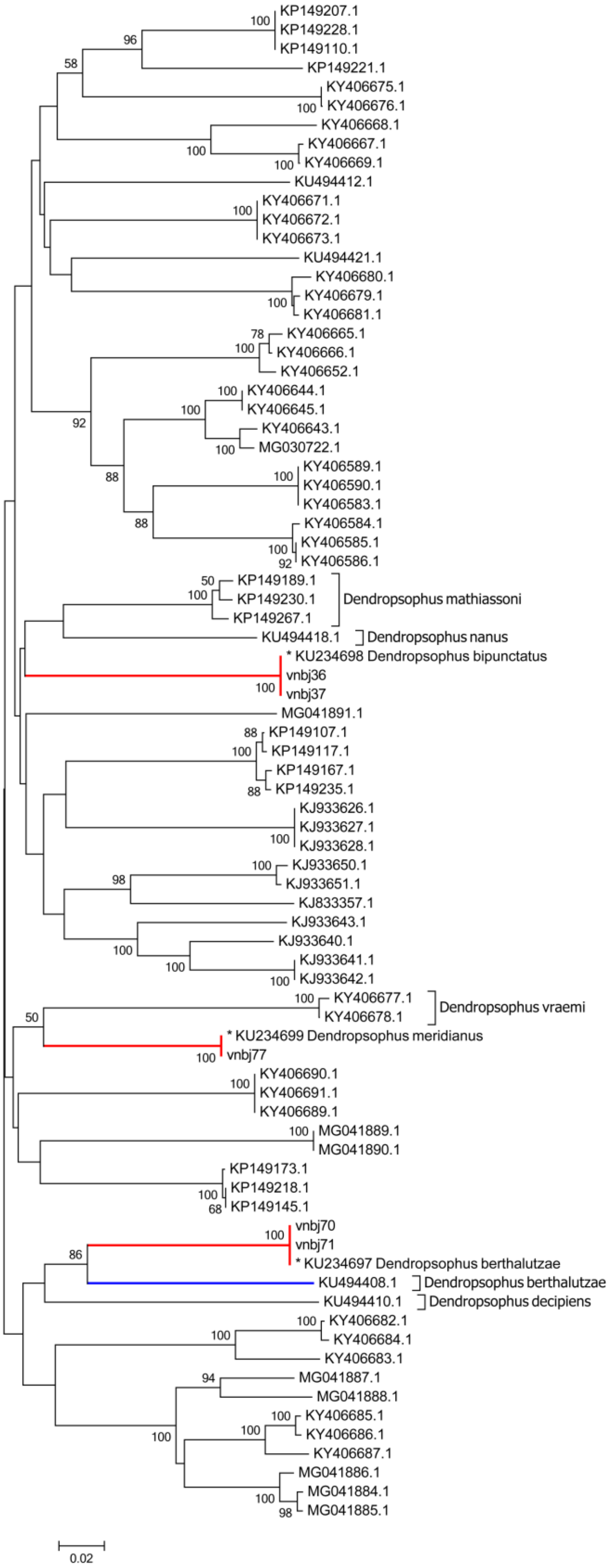

Figure 5 – Neighbor-Joining tree based on the K2P distance of *Dendropsophus meridianus, D. berthalutzae*, and *D. bipunctatus* from the Reserva Ecológica de Guapiaçu, Rio de Janeiro, Brazil, and close relatives. Bootstrap support values over 50% exhibited close to each node. Branches of collected adults and tadpoles specimens in red. Branches for close related specimens of the same species marked in blue.

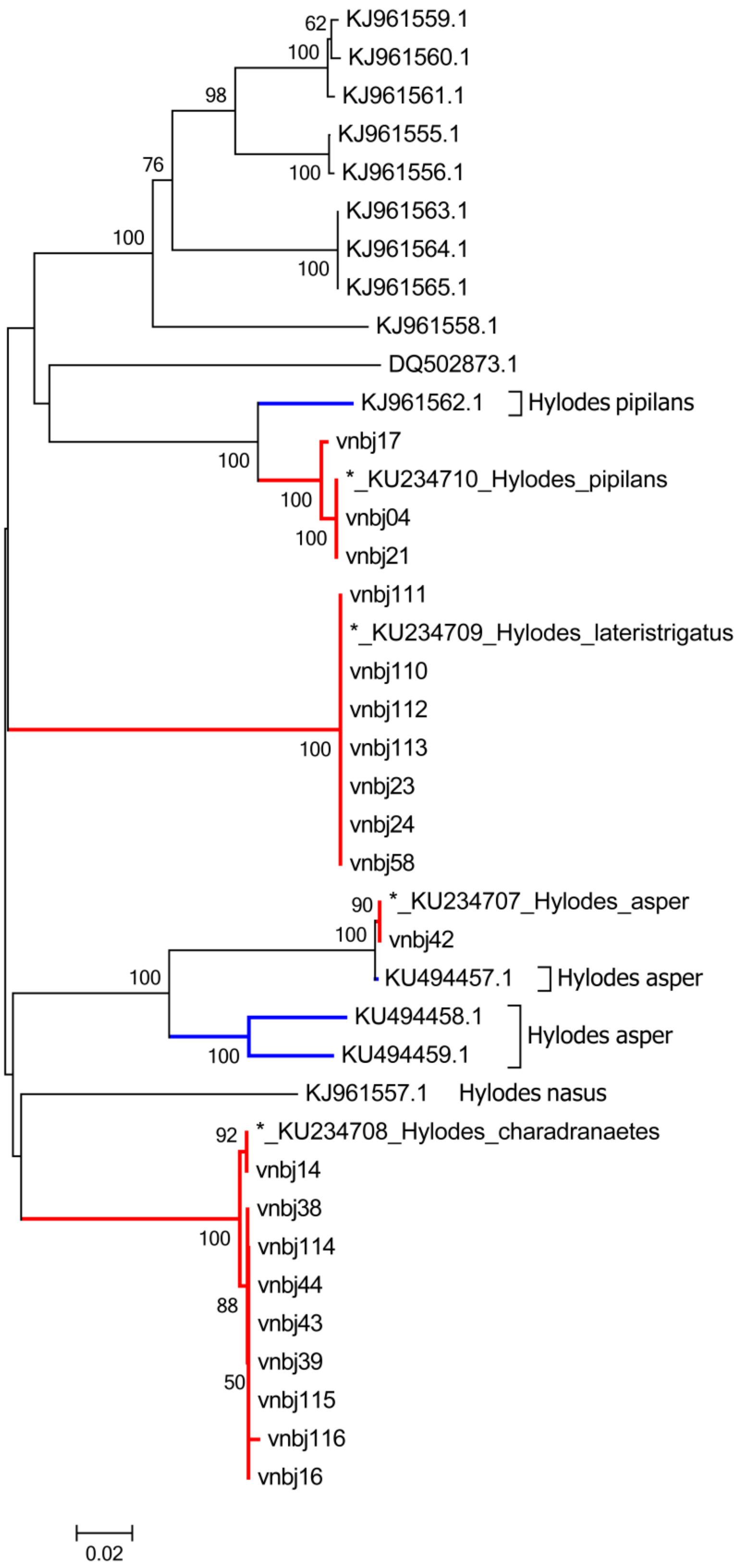

Figure 6 – Neighbor-Joining tree based on the K2P distance of *Hylodes asper, H. pipila,s. H. charadranaetes*, and *H. lateristrigatus* from the Reserva Ecológica de Guapiaçu, Rio de Janeiro, Brazil, and close relatives. Bootstrap support values over 50% exhibited close to each node. Branches of collected adults and tadpoles specimens in red. Branches for close related specimens of the same species marked in blue.

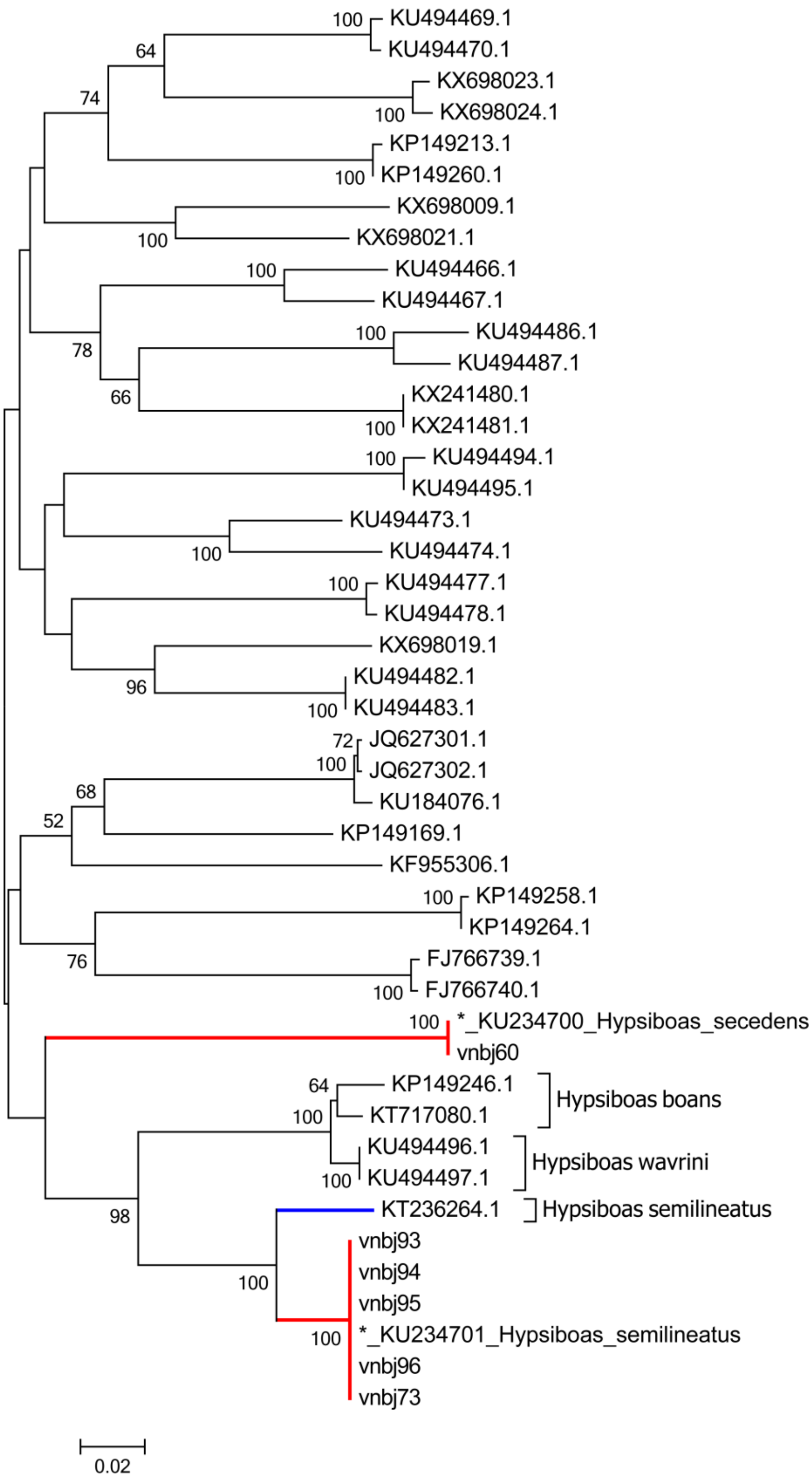

Figure 7 – Neighbor-Joining tree based on the K2P distance of *Hypsiboas secedens*, and *H. semilineatus* from the Reserva Ecológica de Guapiaçu, Rio de Janeiro, Brazil, and close relatives. Bootstrap support values over 50% exhibited close to each node. Branches of collected adults and tadpoles specimens in red. Branches for close related specimens of the same species marked in blue.

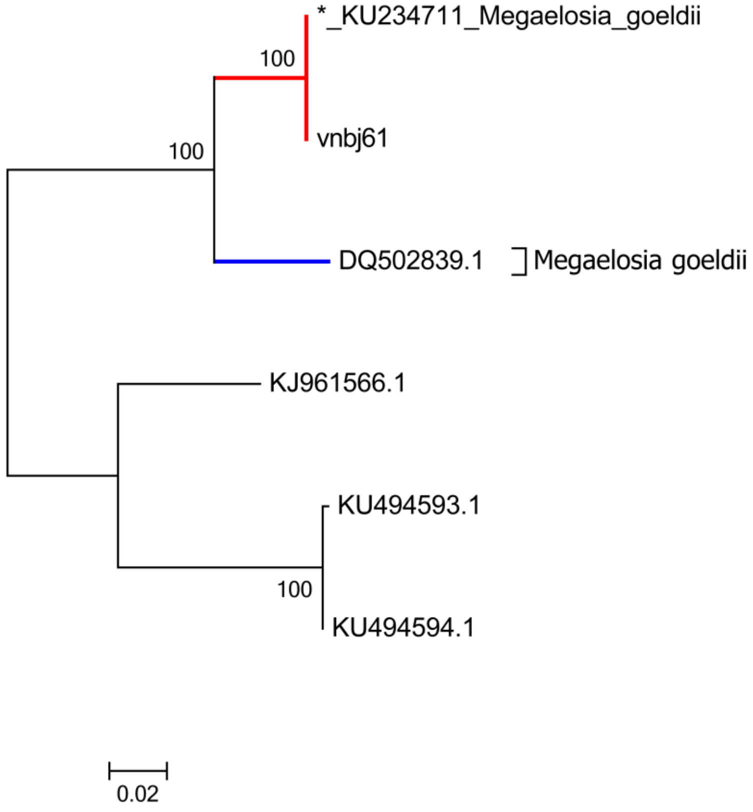

Figure 8 – Neighbor-Joining tree based on the K2P distance of *Megaelosia goeldii* from the Reserva Ecológica de Guapiaçu, Rio de Janeiro, Brazil, and close relatives. Bootstrap support values over 50% exhibited close to each node. Branches of collected adults and tadpoles specimens in red. Branches for close related specimens of the same species marked in blue.

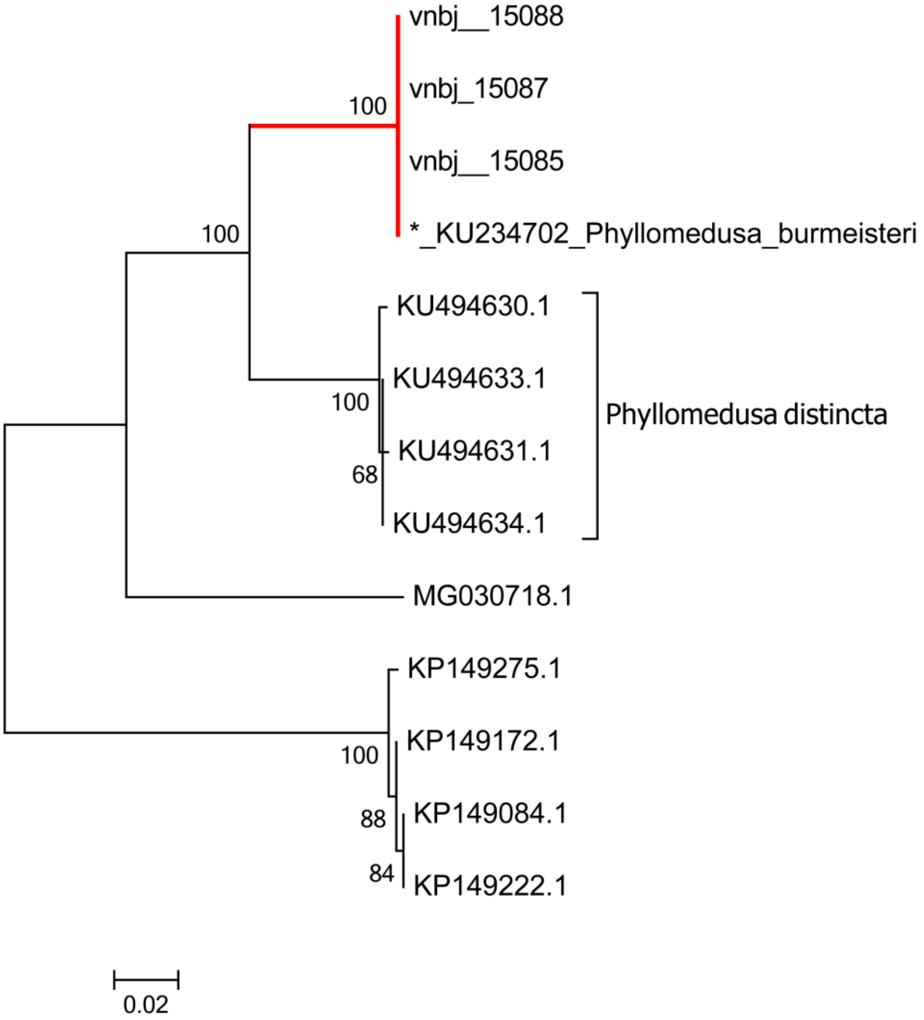

Figure 9 – Neighbor-Joining tree based on the K2P distance of *Phyllomedusa burmeisteri* from the Reserva Ecológica de Guapiaçu, Rio de Janeiro, Brazil, and close relatives. Bootstrap support values over 50% exhibited close to each node. Branches of collected adults and tadpoles specimens in red.

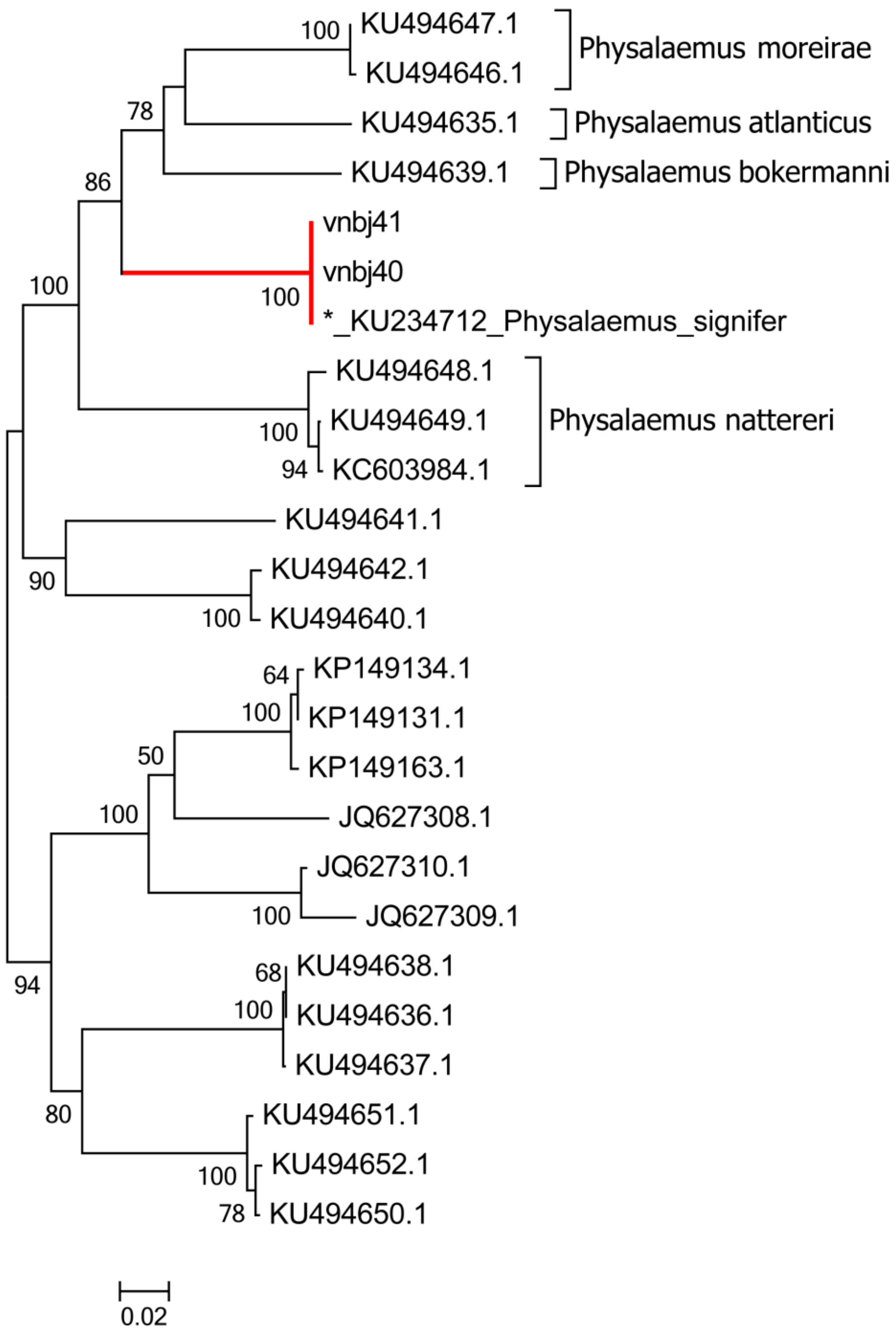

Figure 10 – Neighbor-Joining tree based on the K2P distance of *Physalaemus signifier* from the Reserva Ecológica de Guapiaçu, Rio de Janeiro, Brazil, and close relatives. Bootstrap support values over 50% exhibited close to each node. Branches of collected adults and tadpoles specimens in red.

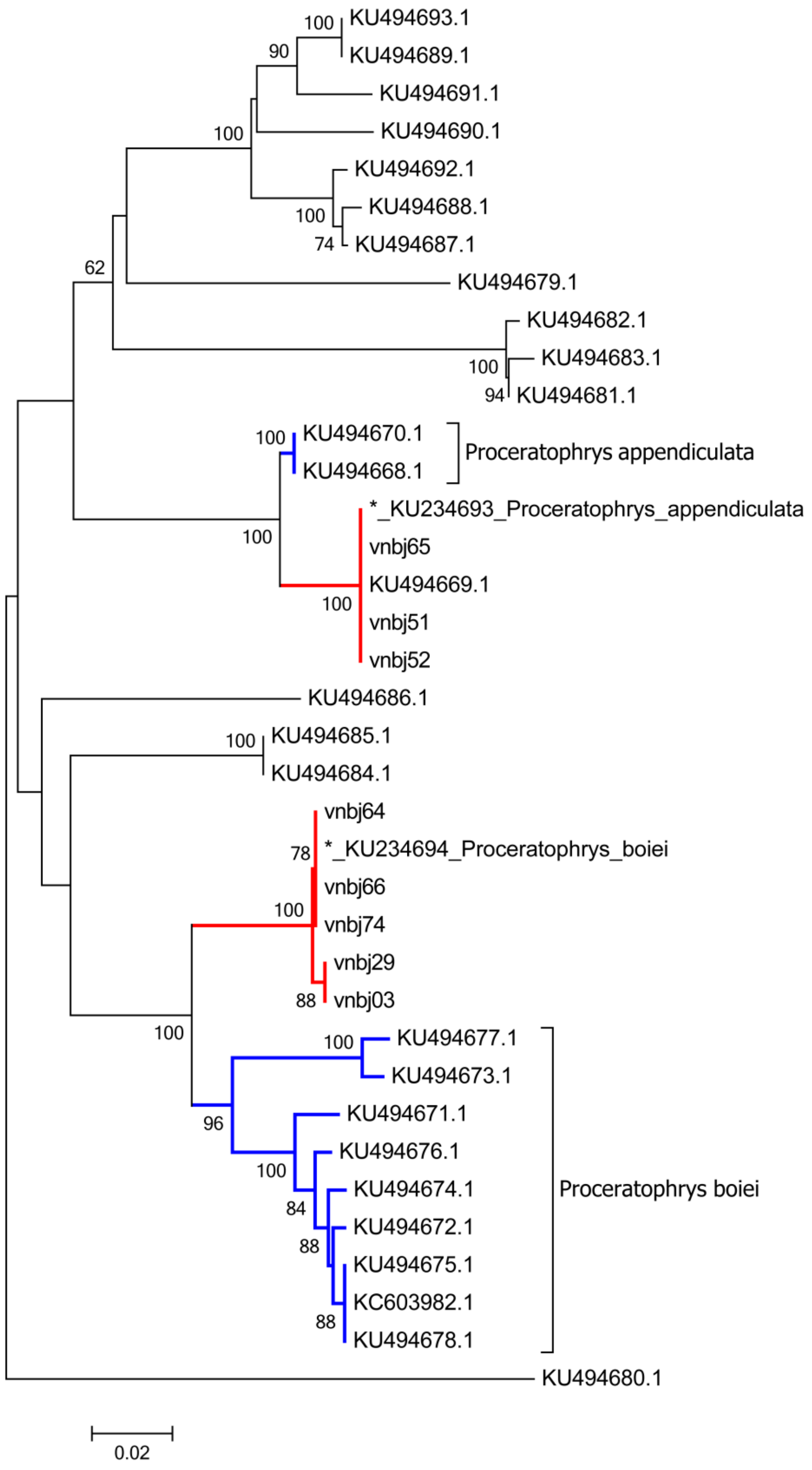

Figure 11 – Neighbor-Joining tree based on the K2P distance of *Proceratophrys appendiculata*, and *P. boiei* from the Reserva Ecológica de Guapiaçu, Rio de Janeiro, Brazil, and close relatives. Bootstrap support values over 50% exhibited close to each node. Branches of collected adults and tadpoles specimens in red. Branches for close related specimens of the same species marked in blue.

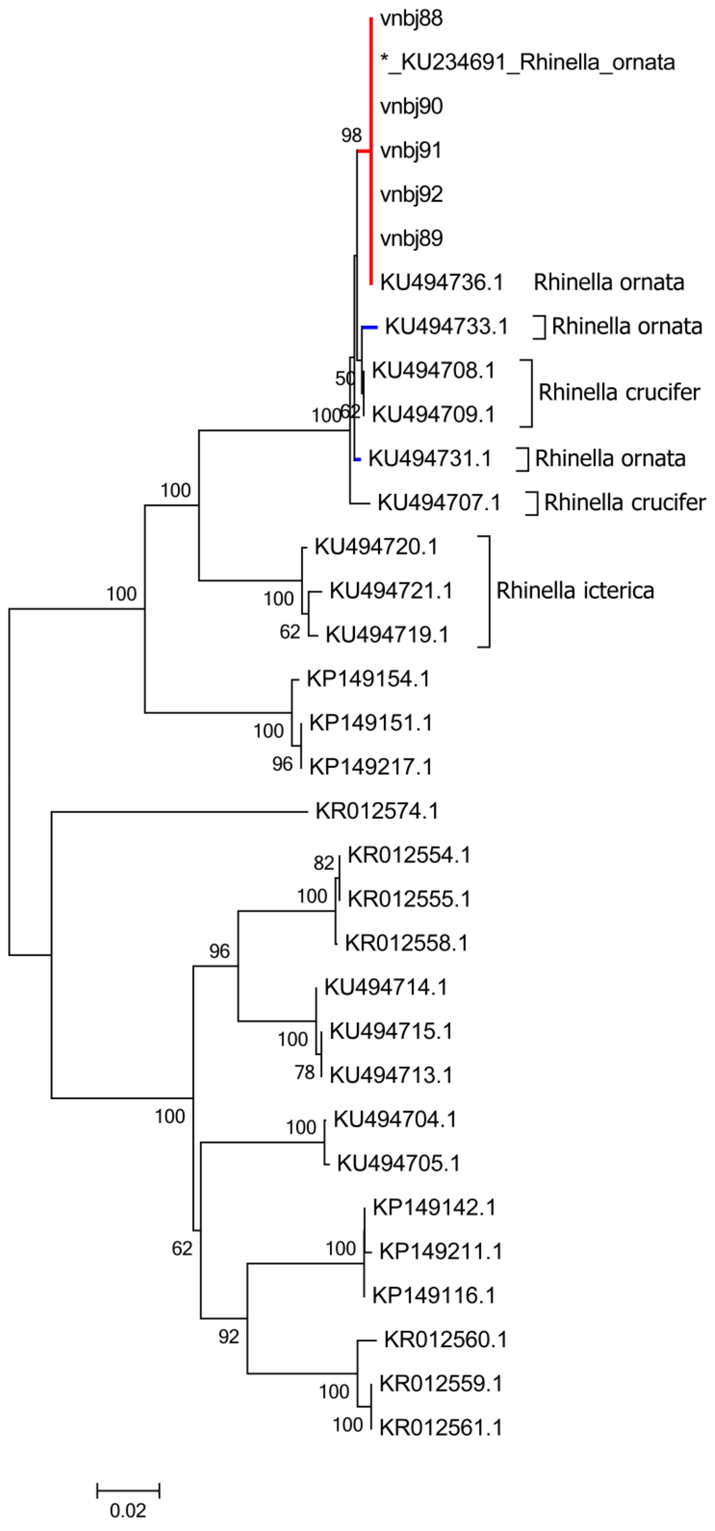

Figure 12 – Neighbor-Joining tree based on the K2P distance of *Rhinella ornata* from the Reserva Ecológica de Guapiaçu, Rio de Janeiro, Brazil, and close relatives. Bootstrap support values over 50% exhibited close to each node. Branches of collected adults and tadpoles specimens in red.

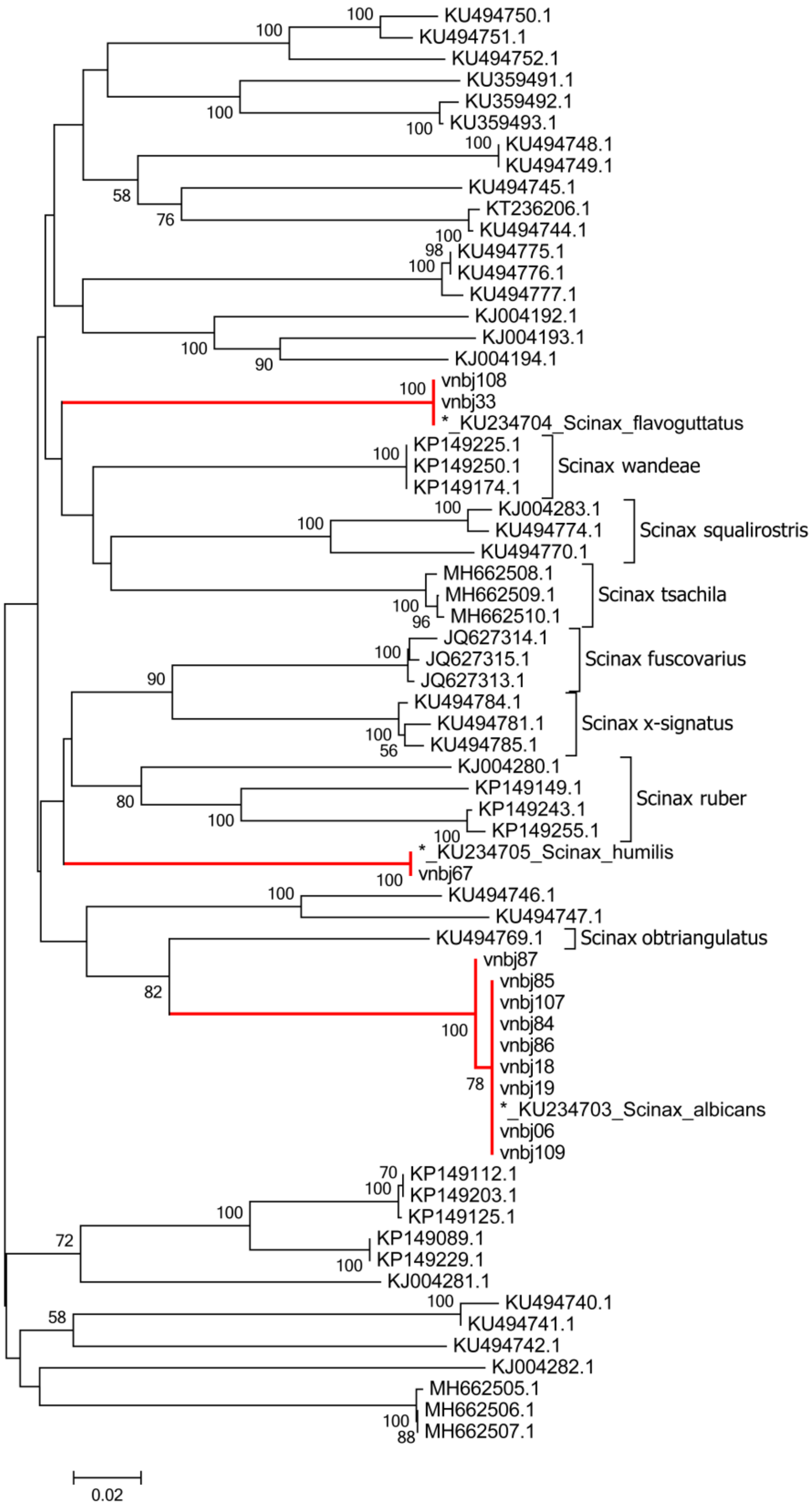

Figure 13 – Neighbor-Joining tree based on the K2P distance of *Synax albican, S. humilis*, and *S. flavoguttatus* from the Reserva Ecológica de Guapiaçu, Rio de Janeiro, Brazil, and close relatives. Bootstrap support values over 50% exhibited close to each node. Branches of collected adults and tadpoles specimens in red.

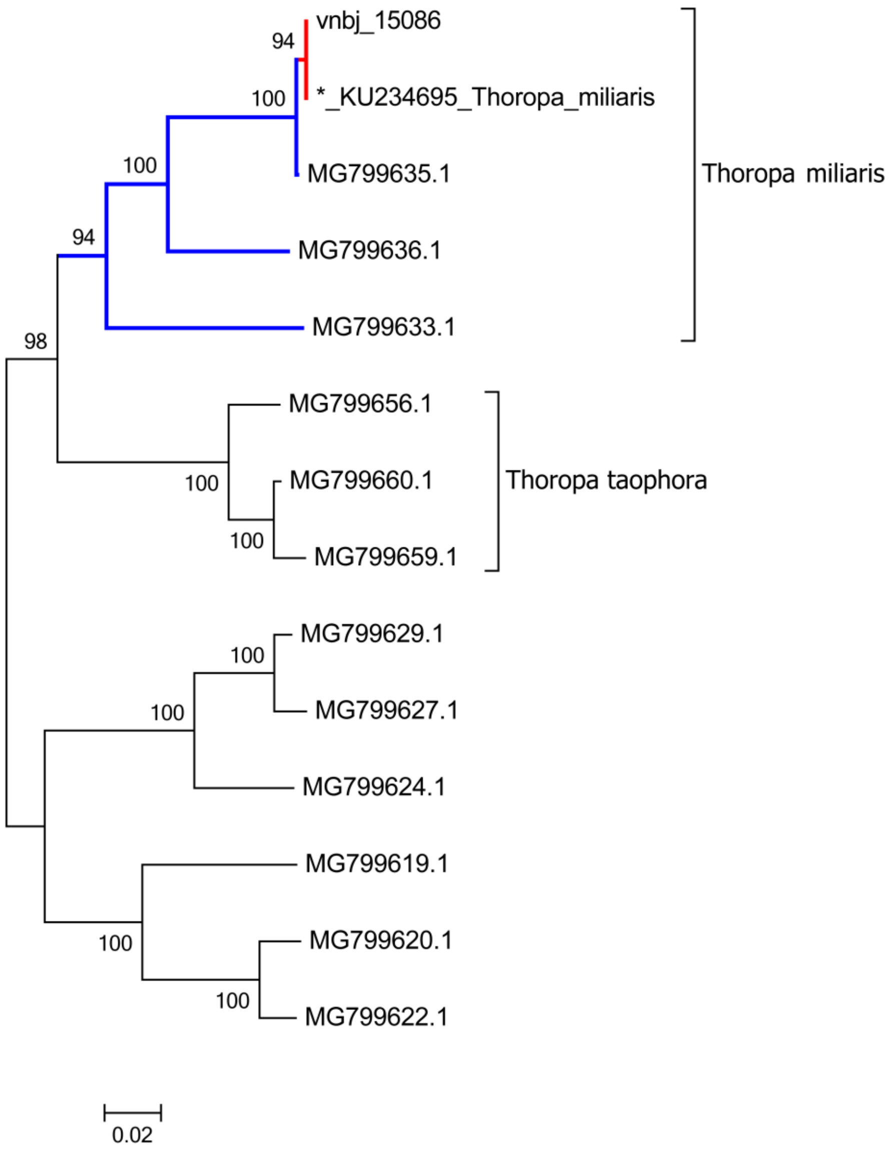

Figure 14 – Neighbor-Joining tree based on the K2P distance of *Thoropa miliaris* from the Reserva Ecológica de Guapiaçu, Rio de Janeiro, Brazil, and close relatives. Bootstrap support values over 50% exhibited close to each node. Branches of collected adults and tadpoles specimens in red. Branches for close related specimens of the same species marked in blue.
