# Supplementary figures and images for: Amphibians on the hotspot: Molecular biology and conservation in the South American Atlantic Rainforest
Source: PLoS One. 2019 Oct 23;14(10):e0224320. doi: 10.1371/journal.pone.0224320 (PMC6808428; doi:10.1371/journal.pone.0224320)

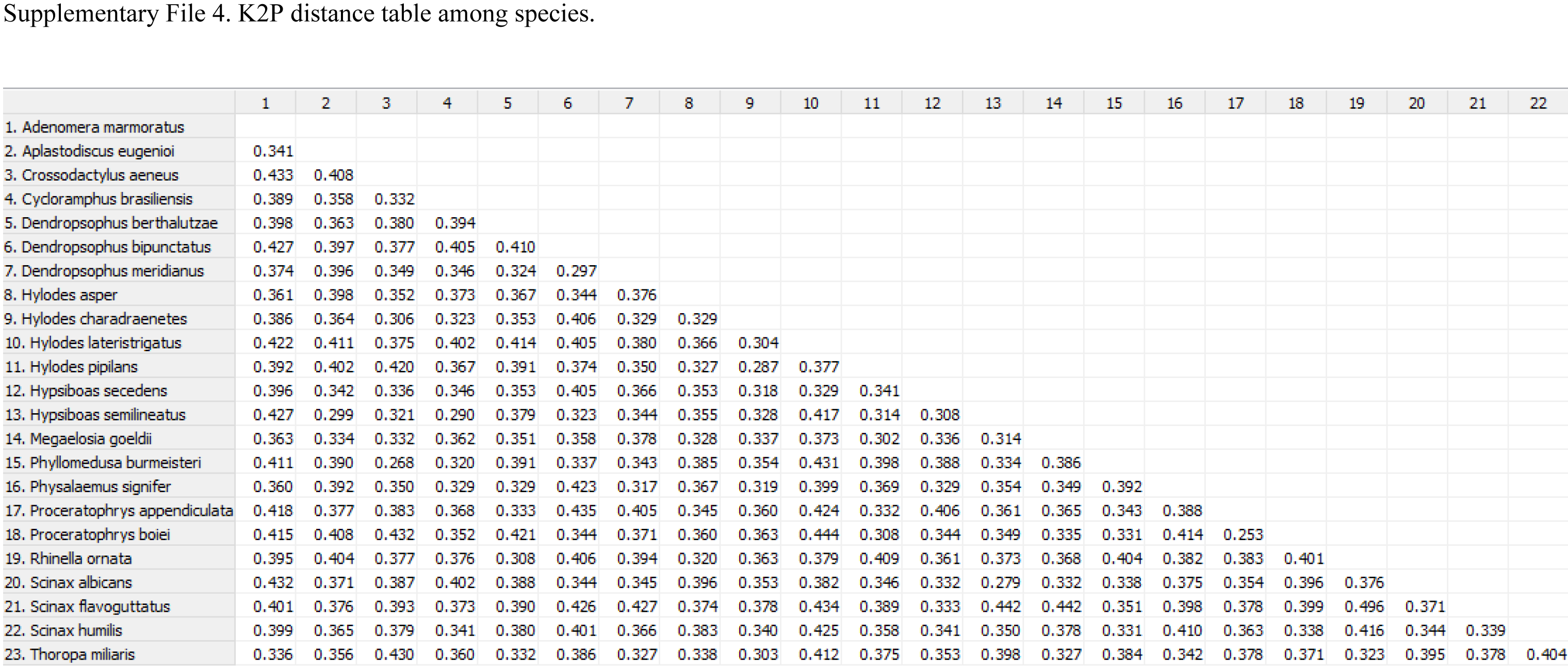

Supplement: S4 File — COI K2P distances for the analyzed groups. (TIF) [file pone.0224320.s004.tif]
