# Supplementary material for: Amphibians on the hotspot: Molecular biology and conservation in the South American Atlantic Rainforest
Source: PLoS One. 2019 Oct 23;14(10):e0224320. doi: 10.1371/journal.pone.0224320 (PMC6808428; doi:10.1371/journal.pone.0224320)
Supplement: S5 File — 16s rRNA distances for the analyzed groups. (PDF) [file pone.0224320.s005.pdf]

Species	N	(1)	(2)	(3)	(4)	(5)	(6)	(7)	(8)	(9)	(10)	(11)	(12)	(13)	(14)	(15)	(16)	(17)	(18)	(19)	(20)	(21)	(22)	(23)
(1) Rhinella ornata	7																							
(2) Cycloramphus brasiliensis	1	0,136																						
(3) Proceratophrys apendiculata	3	0,133	0,100																					
(4) Proceratophrys boiei	4	0,141	0,112	0,039																				
(5) Thoropa miliaris	1	0,108	0,080	0,122	0,120																			
(6) Aplastodiscus eugenioi	5	0,160	0,163	0,146	0,147	0,156																		
(7) Dendropsophus berthalutzae	2	0,143	0,120	0,124	0,117	0,112	0,160																	
(8) Dendropsophus bipunctatus	2	0,143	0,137	0,122	0,120	0,134	0,176	0,097																
(9) Dendropsophus meridianus	3	0,146	0,131	0,120	0,125	0,122	0,166	0,101	0,042															
(10) Hypsiboas secedens	1	0,155	0,143	0,158	0,148	0,126	0,135	0,148	0,164	0,141														
(11) Hypsiboas semilineatus	6	0,143	0,136	0,143	0,139	0,136	0,126	0,151	0,156	0,141	0,106													
(12) Phyllomedusa burmeisteri	3	0,125	0,156	0,129	0,146	0,154	0,185	0,170	0,154	0,156	0,193	0,185												
(13) Scinax albicans	8	0,156	0,146	0,142	0,139	0,131	0,168	0,149	0,146	0,141	0,163	0,165	0,158											
(14) Scinax flavoguttatus	5	0,152	0,151	0,152	0,157	0,139	0,184	0,152	0,149	0,144	0,166	0,169	0,178	0,078										
(15) Scinax humilis	1	0,148	0,134	0,139	0,132	0,121	0,148	0,134	0,142	0,127	0,156	0,148	0,164	0,059	0,091									
(16) Crossodactylus aeneus	13	0,119	0,116	0,130	0,135	0,124	0,165	0,129	0,137	0,124	0,122	0,134	0,149	0,119	0,118	0,122								
(17) Hylodes asper	1	0,136	0,087	0,144	0,144	0,096	0,189	0,142	0,138	0,130	0,141	0,144	0,162	0,143	0,139	0,144	0,107							
(18) Hylodes charadranaetes	8	0,133	0,094	0,120	0,110	0,087	0,172	0,115	0,113	0,101	0,134	0,131	0,144	0,149	0,155	0,139	0,112	0,060						
(19) Hylodes lateristrigatus	7	0,112	0,085	0,108	0,105	0,096	0,151	0,118	0,130	0,111	0,126	0,138	0,145	0,144	0,149	0,139	0,107	0,081	0,061					
(20) Hylodes pipilans	2	0,119	0,096	0,127	0,122	0,094	0,154	0,125	0,127	0,115	0,129	0,134	0,140	0,146	0,144	0,144	0,117	0,078	0,063	0,054				
(21) Megaelosia goeldii	1	0,115	0,082	0,108	0,112	0,092	0,161	0,122	0,132	0,129	0,141	0,128	0,152	0,149	0,149	0,147	0,119	0,076	0,063	0,072	0,069			
(22) Physalemus signifer	2	0,112	0,105	0,138	0,136	0,105	0,150	0,115	0,117	0,117	0,143	0,145	0,134	0,140	0,139	0,126	0,110	0,110	0,105	0,098	0,101	0,110		
(23) Adenomera mamoratus	3	0,156	0,130	0,125	0,137	0,154	0,166	0,132	0,118	0,130	0,172	0,161	0,156	0,154	0,175	0,146	0,154	0,154	0,157	0,137	0,142	0,149	0,134	
